# Trends of socioeconomic inequality in using maternal health care services in Lao People’s Democratic Republic from year 2000 to 2012

**DOI:** 10.1186/s12889-018-5811-0

**Published:** 2018-07-13

**Authors:** Ngan Do, Huong Thi Giang Tran, Alay Phonvisay, Juhwan Oh

**Affiliations:** 1 JW Lee Center for Global Medicine, Seoul National University College of Medicine, 71 Ihwajang-gil, Jongno-gu, Seoul, 13087 Republic of Korea; 2 Department of Global Health, Hanoi Medical University, Hanoi, Vietnam; 3 Department of Economics and Business Management, Graduate Division, National University of Laos, Vientiane, Lao People’s Democratic Republic

**Keywords:** Trends, Inequality, Socioeconomic factors, Maternal health services, Lao People’s Democratic Republic

## Abstract

**Background:**

Socioeconomic inequalities in access to maternal health care have received more attention as it challenges the sustainability of the ongoing achievement in reducing maternal mortality. By promoting access to maternal health care as one of the core targets of the Health Sector Reform, Lao People’s Democratic Republic has reduced maternal mortality dramatically over the last decade. In spite of this improvement, little has been known about the secular trends in disparities of service utilization across different socioeconomic subgroups.

**Methods:**

Two waves of the Multiple Indicator Cluster Survey in the years 2000 and 2012 were pooled for the analysis. We used logistic regression to estimate the likelihood of using antenatal care (ANC) and delivery services with skilled birth attendants (SBA) across different socioeconomic subgroups. Difference-in-difference method was applied to examine the inequality trends across the years by analyzing the interaction terms of the survey years and socioeconomic factors (education, wealth, ethnicity, and residential areas).

**Results:**

Urban-rural disparity was improved over time while there were no educational disparity changes. Rural residential areas showed significant changes than urban areas over time [OR = 2.40; 95% CI: 1.52–3.77 for ANC and OR = 2.16; 95% CI: 1.36–3.42 for SBA]. However, there were aggravations in the disparities between major and minor ethnic group as well as worsening disparities between the rich and poor: i.e. Ethnic minority showed significant aggravation over time [OR = 0.62; 95% CI: 0.44–0.89 for ANC and OR = 0.65; 95% CI: 0.44–0.97 for SBA].

**Conclusions:**

Efforts to increase maternal health service utilization in poor and minority ethnic groups should be emphasized to reduce social inequalities, thus encompassing multiple-sector interventions rather than focusing only on health sector related interventions.

**Electronic supplementary material:**

The online version of this article (10.1186/s12889-018-5811-0) contains supplementary material, which is available to authorized users.

## Background

Maternal mortality remains a major global public health issue, especially in low-middle income countries, despite significant achievements. Between the year 1990 and 2008, the maternal death reduced 34% (from 546,000 to 358,000) and the maternal mortality ratio also declined 34% (from 400 to 260 maternal deaths per 100,000 live births) worldwide [1]. Regardless of this, disparities across countries in reducing maternal mortality is high [2–4]. Sub-Saharan Africa and South Asia are the two regions accounting for 85% of maternal mortality globally [5]. Inequalities in accessing to maternal health care services, quality of care, and social determinants (including education, place of residence, and wealth) are key drivers of the trend [6, 7]. Within countries, women of disadvantaged socioeconomic groups tend to have less access to maternal health care services, receive low quality services, and die from childbirth related causes [8–13]. Furthermore, inequalities challenge the progress of reducing maternal mortality [14, 15]. For example, in Namibia sub-national inequality is one of the main reasons the decreasing maternal mortality trend reversed, with the maternal death rate increasing from 271 (between 1991 and 2000) to 499 deaths per 100,00 live births (between 1998 and 2007) [16].

Lao People’s Democratic Republic (Lao PDR) is one of the ten “fast-track” countries, including Bangladesh, Cambodia, China, Egypt, Ethiopia, Nepal, Peru, Rwanda, and Vietnam, who are doing better than comparable countries in reducing maternal mortality [17]. Maternal mortality ratio has decreased substantially in the last decade, from 1100 (in 1990) to 220 (in 2012) per 100,000 live births [18]. The government has put maternal health care services as the entry point to strengthen the healthcare system in the Health Sector Reform agenda [19]. Various programs have been implemented, including comprehensive health system strengthening interventions in Northern provinces and free deliveries for the poor in Southern provinces. However, research on inequalities of maternal health service utilization among socioeconomic groups in Lao PDR is surprisingly limited. Inadequate research and data pose to significantly challenge addressing the sustainability of the achievements in reducing maternal mortality in the country. Although the differences in the use of maternal services among socioeconomic groups can be found in reports of the government or development partners, the information is given in a very crude level without controlling for other factors.

This paper aims to examine the differences in using maternal health care services across different socioeconomic subgroups in Lao PDR. It also presents the changes in the service utilization patterns and equality trends from year 2000 to 2012.

## Methods

### Data and variables

From the 2000 and 2012, two rounds of the Lao PDR Multiple Indicator Cluster Surveys (MICS) were carried out by the Ministry of Planning and Investment (National Statistical Center) and the Ministry of Health (National Institute of Public Health) with the support of the United Nations International Children’s Emergency Fund (UNICEF). The data was collected using the standard MICS questionnaires and techniques. Eligible participants were defined as women aged 15–49 living as usual residents of the household. They were interviewed with a questionnaire, which consisted of seven parts. Maternal and newborn health was one of sections with more than 20 questions reporting the health care experience of the mother and the newborns. This current study focused on the maternal health care related questions to analyze maternal health care service utilization between the two survey rounds.

The Additional file 1: Table S1 describes the variables. The use of maternal health care services was examined by two outcome variables: Antenatal Care Visit (ANC) and delivery with a Skilled Birth Attendant (SBA). The use of ANC was counted when the women had at least one visit to health professionals during pregnancy. The use of SBA was defined as the delivery experience with a health professional such as a doctor, nurse, midwifed or auxiliary midwife. Traditional birth attendants and community health workers were not considered as health professionals. The socioeconomic equity indicators used in this study were: ethnicity, residential area (urban vs. rural), educational level, and household wealth. Other covariates included mothers’ age and access to media.

### Analysis

We used binary logistic regression to analyze the relationship between outcome variables and socioeconomic factors. The logistic regression Odds Ratios (OR) represents the likelihood of using ANC and delivery by a SBA in each socioeconomic category. It indicates the degree of socioeconomic inequality in utilizing ANC and a SBA of the socioeconomic groups compared to the referenced group. The logistic regression was run on the pooled data of the two rounds of MICS, which allowed us to use the difference-in-difference model to estimate the secular trend. We put two-way interactions between the survey year and the socioeconomic variables (the educational level of the woman, ethnicity, residential area, and household wealth) into the logistic model after adjusting for the mother’s age and exposure to media. The interaction terms helped avoid bias and misinterpretation of the different effects of socioeconomic factors on maternal health care service utilization over time [20–22]. If the interaction between one of socioeconomic variable and survey years was non-significant, the relationship of the outcome variable and the socioeconomic independent variable could be interpreted consistent over time: In other words, there were no changes in the inequality in that independent variable over time. If the interaction term was significant, it should be considered as a change in inequality. In that case, we further present the interaction effects by plotting the interaction into graphs for visual depiction.

## Results

Table 1 summarizes the characteristics of the study sample. More than half of the women in this study were non-Lao. Most of them were in their 20s (53.79% in 2000 and 58.44% in 2012) and 30s (28.79% in 2000 and 24.46% in 2012). Forty-five percent of women reported no education in 2000, which was reduced to 30.67% in 2012. The number of women who ever attended schools increased between 2000 and 2012. Eighty-four percent of women in 2000 reported access to media (radio, newspaper, or television) and in 2012 the number was 75.70%. Eighty one per cent of women were living in rural areas in 2000 which was reduced by 1.24% in 2012. The utilization of ANC and SBAs doubled between the years 2000 and 2012. Twenty-nine percent of the respondents reported accessing to ANC in 2000, in 2012 increased to 51.96%. The rate of delivery with a SBA also increased from 21.23% in 2000 to 38.36% in 2012.

**Table 1:** Description of the study sample

Year	2000	2012
Number of women in the study sample	1161 (100.00%)	4444 (100.00%)
Number of women used antenatal services at least one time	337 (29.05%)	2309 (51.96%)
Number of women delivered with health professionals	246 (21.23%)	1705 (38.36%)
Ethnicity of household head		
Lao	510 (43.97%)	1778 (40.02%)
Non-Lao	651 (56.03%)	2666 (59.98%)
Mother’s age		
15–19	138 (11.90%)	580 (13.05%)
20–29	625 (53.79%)	2597 (58.44%)
30–39	334 (28.79%)	1087 (24.46%)
40–49	64 (5.52%)	180 (4.05%)
Mother’s education		
None	526 (45.27%)	1363 (30.67%)
Primary	451 (38.86%)	1855 (41.74%)
Secondary and higher	184 (15.87%)	1226 (27.59%)
Access to media (radio, newspaper, TV)		
Yes	975 (84.01%)	3364 (75.70%)
No	186 (15.99%)	1080 (24.30%)
Residential area		
Urban	218 (18.79%)	890 (20.03%)
Rural	943 (81.21%)	3554 (79.97%)
HH wealth index quintile		
Poorest quintile	313 (26.98%)	1367 (30.76%)
Second quintile	247 (21.30%)	1010 (22.73%)
Third quintile	210 (18.10%)	848 (19.08%)
Fourth quintile	203 (17.50%)	668 (15.03%)
Richest quintile	187 (16.12%)	551 (12.40%)

Table 2 presents the differences in maternal health care service utilization across different socioeconomic groups. Women living in households headed by non-Lao ethnics were less likely to use both ANC [OR = 0.56; 95% CI: 0.47–0.66] and SBA [OR = 0.71; 95% CI: 0.59–0.85]. Compared to the mothers who had never attended school, the mothers who had attended primary school were more likely to use ANC [OR = 2.32; 95% CI: 1.97–2.74] and more likely delivery with a SBA [OR = 1.40; 95% CI: 1.12–1.70]. Women with secondary or higher education were more likely to use ANC [OR = 4.58; 95% CI: 3.70–5.68] and more likely to use a SBA [OR = 3.71; 95% CI: 2.96–4.65]. There were statistically significant differences in the service use across residential areas. The women living in rural areas were less likely to use ANC [OR = 0.36; 95% CI: 0.29–0.43] and a SBA [OR = 0.29; 95% CI: 0.24–0.35]. The richest quintile women were more likely to use antenatal services [OR = 3.41; 95% CI: 2.53–4.59] and more likely to deliver with a SBA [OR = 8.21; 95% CI: 5.99–11.27] compared to the poorest quintile women. The likelihood of using maternal services was lower among those who had no access to media such as newspaper, radio, or television.

**Table 2:** The association between socio-demographic factors and maternal health service utilization in Lao PDR (2000, 2012)

	ANC		SBA	
	OR	95% CI	OR	95% CI
HH head ethnic (ref. Lao)				
Non-Lao ethnic	0.56^c^	0.47–0.66	0.71^c^	0.59–0.85
Education (ref. no schooling)				
Primary	2.32^c^	1.97–2.74	1.40^c^	1.12–1.70
Secondary and higher	4.58^c^	3.70–5.68	3.71^c^	2.96–4.65
Residential Area (ref. urban)				
Rural	0.36^c^	0.29–0.43	0.29^c^	0.24–0.35
Wealth (ref. poorest quintile)				
Second quintile	1.81^c^	1.50–2.18	2.22^c^	1.76–2.80
Third quintile	2.37^c^	1.92–2.92	3.58^c^	2.80–4.57
Fourth quintile	2.75^c^	2.16–3.51	5.07^c^	3.87–6.63
Richest quintile	3.41^c^	2.53–4.59	8.21^c^	5.99–11.27
Age (ref. age group:15–19)				
20–29	1.27^b^	1.04–1.55	0.82	0.66–1.01
30–39	1.13	0.90–1.42	0.80^*^	0.61-0.99
40–49	0.87	0.59–1.27	0.68	0.44–1.04
Access to media (ref. ever access to media)				
Non media access	0.51^c^	0.43–0.62	0.70^b^	0.56–0.88
Province (ref. Vientiane Capital)				
Phongsaly	0.32^c^	0.19–0.56	0.35^c^	0.20–0.61
Luangnamtha	1.45	0.84–2.51	0.89	0.52–1.52
Oudomxay	0.61^a^	0.37-0.99	0.46^b^	0.28–0.76
Bokeo	0.65	0.39–1.08	0.76	0.46–1.25
Huaphanh	0.53^a^	0.33-0.87	0.58^a^	0.36-0.94
Luangprabang	0.72	0.44–1.17	0.44^b^	0.26–0.72
Xayabury	1.14	0.69–1.90	0.46^b^	0.28–0.75
Xiengkhuang	0.69	0.42–1.16	0.57^a^	0.35-0.87
Vientiane province	1.04	0.63–1.72	0.54^a^	0.34-1.14
Borikhamxay	0.53^a^	0.32-0.89	0.70	0.43–1.14
Khammuane	0.46^b^	0.29–0.75	0.45^b^	0.28–0.72
Savannakhet	0.57^a^	0.36-0.90	0.54^b^	0.35–0.83
Saravane	0.77	0.48–1.22	0.65	0.41–1.01
Sekong	0.63	0.38–1.01	0.41^c^	0.25–0.67
Champasak	0.30^c^	0.19–0.48	0.33^c^	0.22–0.51
Attapeu	0.49^b^	0.30–0.82	0.19^c^	0.11–0.33
Year (ref. year 2000)				
2012	1.11^c^	1.09–1.12	1.10^c^	1.08–1.11
Constant	2.41e-88	7.8e-102-7.4e-75	4.05e-80	3.3e-95-4.96e-65

^a^: *p*-value<=0.05; ^b^: *p*-value<=0.01; ^c^: *p*-value<=0.001

**Fig. 1:**
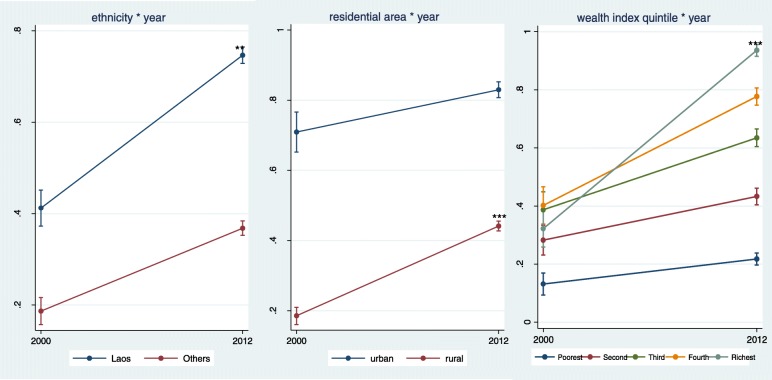
Secular trend of antenatal service utilization 2000–2012

**Fig. 2:**
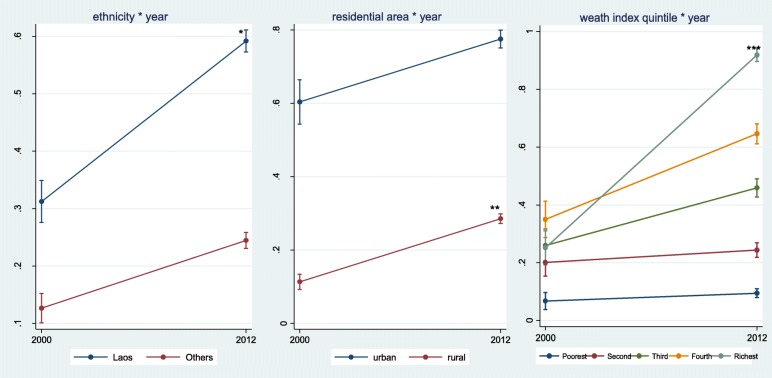
Secular trend of delivery with health professionals 2000–2012

Table 3, Fig. 1, and Fig. 2 show secular trend of diverse socioeconomic inequalities through the interaction effects of socioeconomic factors and survey year. The interaction between educational attainment of the mothers and the survey year was not statistically significant. Statistical significance was found in the interaction terms of survey year and ethnicity [OR = 0.62; 95% CI: 0.44–0.89 for ANC and OR = 0.65; 95% CI: 0.44–0.97 for SBA]. Survey year and residential areas also showed statistically significant changes [OR = 2.40; 95% CI: 1.52–3.77 for ANC and OR = 2.16; 95% CI: 1.36–3.42 for SBA]. The plots in Fig. 1 and Fig. 2 showed an increase in using both ANC and a SBA. The increase in ANC and delivery by a SBA were higher among Lao ethnic woman than non-Lao, among rural residents than urban, and among the richer women compared to the poorer women.

**Table 3:** Interaction terms between socio-economic characteristics and years of survey for maternal service utilization

	ANC		SBA	
	OR	95% CI	OR	95% CI
Ethnicity of household head (ref. Lao)				
Non-Lao	0.62^b^	0.44–0.89	0.65^a^	0.44-0.97
Mother’s education (ref. no education)				
Primary	1.15	0.76–1.75	1.21	0.75–1.95
Secondary and higher	0.68	0.40–1.15	1.09	0.64–1.86
Residential area (ref. urban)				
Rural	2.40^c^	1.52–3.77	2.16^b^	1.36–3.42
HH wealth index quintile (ref. the poorest quintile)				
Second quintile	0.89	0.51–1.53	0.79	0.40–1.53
Third quintile	0.99	0.57–1.7	1.27	0.64–2.49
Fourth quintile	1.28	0.73–2.26	1.23	0.63–2.38
Richest quintile	3.85^c^	1.99–7.43	6.41^c^	3.07–13.38

^a^: *p*-value<=0.05; ^b^: *p*-value<=0.01; ^c^: *p*-value<=0.001

## Discussion

This research explored the secular trend of inequalities in Lao maternal health care services. Urban-rural disparity improved over time while there were no educational disparity changes. However, there were aggravation in the disparities between major and minor ethnic group as well as in the disparities between the rich and poor. The use of ANC and SBA services almost doubled from the year 2000 to 2012 in Lao PDR even though the utilization of these services were still low, compared to other MDG5 fast-track countries. About half of Lao women use ANC services while 89% in Cambodia, 88% in Vietnam, and 98% in Rwanda utilize ANC. [23–26]. The rate of delivery assisted by skilled birth attendant in Lao PDR is 38% while it is 71% in Cambodia, 84% in Vietnam, and 69% in Rwanda [25].

This study has some limitations that should be noted. First, the data collected are from respondents’ recall information, which was not validated with other objective data sources such as health facilities’ ANC and SBA registration data. The validity of self-reported data constitutes some concerns in the literature. When the recall period is long, the mother might not remember all the events and activities she experienced, especially when she had several deliveries during the recall period. Second, the study relies on cross-sectional data with potential inconsistency between the questionnaires. The survey in 2000 did not collect information on postnatal care while the survey in 2012 had this information. As a result, the study could not assess all the three maternal health service utilization variables (antenatal, delivery, and postnatal care services). Therefore, the study focused only on two outcome variables: antenatal services and delivery with health professionals. The inconsistencies between the questionnaires also lead to the third limit of this study. We did not have enough information to control for other household and community factors that could influence the outcome variables such as marital status, husband’s education, availability of maternal health services within the residential areas, and the distance from the women’s house to the nearest health facilities.

Over the last decade, the number of women living in urban areas increased. The catch-up increase in using ANC is higher among women living in rural areas even though women living in urban areas are still using more services. This might be attributed to the interventions of the government and development partners as these programs have been focusing more on rural than urban areas. However, ethnic disparities in the use of services increased across ethnic groups. Lao ethnic women used more maternal health care services than non-Lao women. The increase in the use of services over time was higher among Lao ethnic women, increasing the gap between major and minor ethnic groups. This result may reflect the influence of culture on the maternal health care seeking behaviors. Many minor ethnicities in Lao PDR believe that the practice of child bearing is the responsibility of the mother herself and she should not receive any assistance from other people [27, 28]. Women with higher education are more likely to use maternal health services, it is unclear whether this trend is the same among non-Lao women. Although the exposure to media help increase the use of maternal health care services, over the last decade the media exposure rate decreased by 9%. Such a decrease might be explained by the survey questions as the survey only asked the women about their exposure to radio, newspaper, and television and did not include the internet and social media. Therefore, further studies on the influence of education and media on the changes in maternal health care service seeking behavior are necessary, especially among minority ethnic groups and in rural areas.

## Conclusions

Lao PDR has made dramatic progress over the last decade in reducing maternal mortality and became one of the 10 fast-track countries to achieve MDG 5. However, the utilization rate of maternal health care services should be improved to catch up with other fast-track countries. Moreover, the inequalities in utilizing maternal health care services across several different socioeconomic groups are also apparent. Therefore, in order to sustain the current progress and further reduce maternal mortality, the socioeconomic inequality issues should be addressed more aggressively. The improvement in disparities between rural and urban areas should go further for the most difficult remote areas in the high mountains where most minority people are living. Apart from health-related interventions, multi-sector participation, such as education and media, is needed to catch up with the socioeconomic changes and their effects on maternal health care services in Lao PDR.

## Additional file

Additional file 1: **Table S1.** Summary of the variables used in the study. All the variables in the model of the study are summarized in this supplementary file. (DOCX 15 kb)

## Abbreviations

ANC: Antenatal care
CI: Confidence intervals
Lao PDR: Lao Peoples’ Democratic Republic
MDG: Millennium Development Goal
MICS: Multiple Indicator Cluster Survey
OR: Odds ratio
SBA: Skilled birth attendant
UNICEF: United Nations International Children’s Emergency Fund

## References

[1] Zureick-Brown S, Newby H, Chou D, Mizoguchi N, Say L, Suzuki E, et al. Understanding global trends in maternal mortality. Int Perspect Sex Reprod Health. 2013;39:32–41. 10.1363/3903213 PMC3886625 23584466

[2] Houweling TA, Ronsmans C, Campbell OM, Kunst AE (2007). Huge poor-rich inequalities in maternity care: an international comparative study of maternity and child care in developing countries. Bull World Health Organ.

[3] Say L, Raine R (2007). A systematic review of inequalities in the use of maternal health care in developing countries: examining the scale of the problem and the importance of context. Bull World Health Organ.

[4] Alam N, Hajizadeh M, Dumont A, Fournier P (2015). Inequalities in maternal health care utilization in sub-Saharan African countries: a multiyear and multi-country analysis. PLoS One.

[5] Mezmur M, Navaneetham K, Letamo G, Bariagaber H (2017). Individual, household and contextual factors associated with skilled delivery care in Ethiopia: evidence from Ethiopian demographic and health surveys. PLoS One.

[6] Ahmed S, Creanga AA, Gillespie DG, Tsui AO (2010). Economic status, education and empowerment: implications for maternal health service utilization in developing countries. PLoS One.

[7] Babalola S, Fatusi A (2009). Determinants of use of maternal health services in Nigeria--looking beyond individual and household factors. BMC Pregnancy Childbirth.

[8] Hajizadeh M, Alam N, Nandi A (2014). Social inequalities in the utili zation of maternal care in Bangladesh: have they widened or narrowed in recent years?. Int J Equity Health.

[9] Hong R, Them R (2015). Inequality in access to health Care in Cambodia. Asia Pacific J Public Heal.

[10] Anwar I. Inequity in maternal health-care in Bangladesh. Bull World Health Organ. 2008;86 http://www.who.int/bulletin/volumes/86/4/07-042754.pdf. Accessed 4 Oct 2017 10.2471/BLT.07.042754 PMC2647426 18438513

[11] Bonfrer I, van de Poel E, Grimm M, Van Doorslaer E (2014). Does the distribution of healthcare utilization match needs in Africa?. Health Policy Plan.

[12] Obiyan MO, Kumar A (2015). Socioeconomic inequalities in the use of maternal health Care Services in Nigeria. Sage Open.

[13] Pathak PK, Singh A, Subramanian SV (2010). Economic inequalities in maternal health care: prenatal care and skilled birth attendance in India, 1992–2006. PLoS One.

[14] Boutayeb A, Helmert U (2011). Social inequalities, regional disparities and health inequity in north African countries. Int J Equity Health.

[15] McKinnon B, Harper S, Kaufman JS, Bergevin Y (2014). Socioeconomic inequality in neonatal mortality in countries of low and middle income: a multicountry analysis. Lancet Glob Heal.

[16] Zere E, Tumusiime P, Walker O, Kirigia J, Mwikisa C, Mbeeli T (2010). Inequities in utilization of maternal health interventions in Namibia: implications for progress towards MDG 5 targets. Int J Equity Health.

[17] The Partnership for Maternal (2014). Newborn & Child Health. The PMNCH 2013 progress report.

[18] UNDP. MDGs Progress Report Lao PDR 2013 | UNDP in Lao PDR. 2014. http://www.la.undp.org/content/lao_pdr/en/home/library/mdg/mdgs-progress-report-lao-pdr-2013.html. Accessed 3 Oct 2017.

[19] Health M of. Health sector reform by 2020. Vientiane; 2013. chrome-extension://oemmndcbldboiebfnladdacbdfmadadm/http://www.nationalplanningcycles.org/sites/default/files/planning_cycle_repository/lao_peoples_democratic_republic/lao_hsr_strategy_and_framework_2025_english_final_201702.pdf. Accessed 3 Oct 2017.

[20] Vatcheva KP, Lee M, McCormick JB, Rahbar MH (2015). The effect of ignoring statistical interactions in regression analyses conducted in epidemiologic studies: an example with survival analysis using cox proportional hazards regression model. Epidemiol (Sunnyvale).

[21] Bauer DJ, Curran PJ (2005). Probing interactions in fixed and multilevel regression: inferential and graphical techniques. Multivariate Behav Res.

[22] Ai C, Norton EC (2003). Interaction terms in logit and probit models. Econ Lett.

[23] Options Consultancy Services/Evidence for Action (2013). Cambridge economic policy associates TP for MN. Success factors for Women’s and Children’s health: country specific review of data and literature on 10 fast-track countries’ progress towards MDGs 4 and 5 an input to the country policy analyses and multistakeholder review meetings.

[24] Dingle A, Powell-Jackson T, Goodman C (2013). A decade of improvements in equity of access to reproductive and maternal health services in Cambodia, 2000-2010. Int J Equity Health.

[25] Kuruvilla S, Schweitzer J, Bishai D, Chowdhury S, Caramani D, Frost L (2014). Success factors for reducing maternal and child mortality. Bull World Health Organ.

[26] World health statistics 2012. Geneva, Switzerland http://www.who.int/gho/publications/world_health_statistics/EN_WHS2012_Full.pdf. Accessed 5 Oct 2017.

[27] King EM, Van De Walle D, Bank W. Laos: ethno-linguistic diversity and disadvantage. 2010. http://siteresources.worldbank.org/EDUCATION/Resources/278200-1121703274255/1439264-1288632678541/7520452-1292532951964/Session2_Walle_Laos_Dec.20.pdf. Accessed 3 Oct 2017.

[28] Ngan DK, Kang M, Lee C, Vanphanom S (2016). “Back to basics” approach for improving maternal health care services utilization in Lao PDR. Asia Pacific J Public Heal.

