# Supplementary material for: Trends of socioeconomic inequality in using maternal health care services in Lao People’s Democratic Republic from year 2000 to 2012
Source: BMC Public Health. 2018 Jul 13;18:875. doi: 10.1186/s12889-018-5811-0 (PMC6045842; doi:10.1186/s12889-018-5811-0)
Supplement: Supplementary file 1 — Table S1. Summary of the variables used in the study. All the variables in the model of the study are summarized in this supplementary file. (DOCX 15 kb) [file 12889_2018_5811_MOESM1_ESM.docx]

Table S1. Summary of the variables used in the study

| Variable name | Definition |
| --- | --- |
| Antenatal care (ANC) | [Binary, dependent variable]  = 0 if the mother had no antenatal care visit to health professionals;  = 1 if the mother at least once visited the doctor, nurse, midwife, or auxiliary midwife. Traditional birth attendance and community health worker are not considered as health professionals. |
| Skilled birth attendance (SBA) | [Binary, dependent variable]  = 0 if the mother did not receive help from health professionals at delivery;  = 1 if the mother received help from doctor, nurse, midwife, or auxiliary midwife at delivery. Traditional birth attendance and community health worker are not considered as health professionals. |
| Ethnicity | [Binary, independent variable]  = 1 if the head of the household where the woman is living is Lao ethnic;  = 2 if the ethnic is not Lao |
| Age | [Four categories, independent variable]  = 1 if the woman’s age is in the range [15-19]  = 2 if the woman’s age is in the range [20-29]  = 3 if the woman’s age is in the range [30-39]  = 4 if the woman’s age is in the range [40-49] |
| Education | [Three categories, independent variable]  = 1 if the woman never went to school  = 2 if the woman highest educational level is primary school  = 3 if the woman highest educational level is secondary school or higher level school |
| Access to media | [Binary, independent variable]  = 0 if the woman never access to newspaper or radio or television  = 1 if the woman ever access to newspaper or radio or television |
| Residential area | [Binary, independent variable]  = 0 if the woman lives in rural areas  = 1 if the woman lives in urban areas |
| Household Wealth | [Five categories, independent variable]: was calculated using household assets data collected from MICS survey. These assets or consumer items consists of durable consumer goods (such as television, fridge, air-conditioner, washing machine), dwelling characteristics (such as material of floor roof-top, source of drinking water), ownership of land and agriculture tools (such as animal-drawn cart, boat with motor)  = 1 if the woman is living in the household of the poorest quintile  = 2 if the woman is living in the household of the second quintile  = 3 if the woman is living in the household of the middle quintile  = 4 if the woman is living in the household of the fourth quintile  = 5 if the woman is living in the household of the richest quintile |
